# Non-random pre-transcriptional evolution in HIV-1. A refutation of the foundational conditions for neutral evolution

**DOI:** 10.1590/S1415-47572009005000025

**Published:** 2009-01-30

**Authors:** Carlos Y Valenzuela

**Affiliations:** Programa Genética Humana, Instituto de Ciencias Biomédicas, Facultad de Medicina, Universidad de Chile, SantiagoChile

**Keywords:** non-random evolution, HIV-1, Bose-Einstein distribution, pre-transcriptional evolution, non-random sequences

## Abstract

The complete base sequence of HIV-1 virus and GP120 *ENV* gene were analyzed to establish their distance to the expected neutral random sequence. An especial methodology was devised to achieve this aim. Analyses included: a) proportion of dinucleotides (signatures); b) homogeneity in the distribution of dinucleotides and bases (isochores) by dividing both segments in ten and three sub-segments, respectively; c) probability of runs of bases and No-bases according to the Bose-Einstein distribution. The analyses showed a huge deviation from the random distribution expected from neutral evolution and neutral-neighbor influence of nucleotide sites. The most significant result is the tremendous lack of CG dinucleotides (p < 10^-50^ ), a selective trait of eukaryote and not of single stranded RNA virus genomes. Results not only refute neutral evolution and neutral neighbor influence, but also strongly indicate that any base at any nucleotide site correlates with all the viral genome or sub-segments. These results suggest that evolution of HIV-1 is pan-selective rather than neutral or nearly neutral.

## Introduction

The Neutral Theory of evolution is mostly based on the emergence of new alleles or nucleotide bases by random mutation and their subsequent random fixation, loss or polymorphic maintenance (Kimura, 1968; King and Jukes, 1969; Crow and Kimura, 1970; Kimura, 1979, 1991, 1993). Kimura (1957) based this theory on the random fluctuation of gene frequencies described by stochastic matrices or by the mathematics of Brownian motion. He followed the development performed by Wright (1931) and Feller (1951), and applied Kolmogorov forward and backward equations (Crow and Kimura, 1970) elaborated for dealing with random motion to describe the random variation of allele frequencies, in order to predict the random pathway of a new mutant allele.

Studies on codon usage, synonymous and non-synonymous substitutions made the pure neutralism untenable, so it was replaced by nearly neutral evolution (Kreitman, 1996a, 1996b; Ohta, 1996; Hey, 1999). The status of the Neutral Theory has been extensively revised, and it is considered mostly refuted by phylogenetic analyses of codons, synonymous and non-synonymous substitutions and the different evolutionary behavior of the 1^st^, 2^nd^ and 3^rd^ codon position (Nei, 2005). Most of, if not all, studies performed to test neutral versus selective evolution compare amino acid or nucleotide (involved in protein synthesis, post-transcriptional events) variations among individuals or taxa. These studies cannot solve the evolutionary condition of the genetic code itself, the acquisition and maintenance of genetic codes, pre-transcriptional evolution, genome sizes, maintenance of nucleotide isochores and signatures, chromosomal features, replication velocities, non-coding DNA and several other genome traits not related to transcription. Also, these analyses cannot inform on selective processes underlying pure base sequences and those related to the origin of life when the four bases and genetic code were established; they are blind for the most important part of evolution (Valenzuela, 2002a). Moreover, these studies have epistemic circularities from which they cannot go out. Studies on synonymous or non-synonymous substitutions assume, without demonstration (creating a circular tautology), that synonymous substitutions are neutral or less selective than non-synonymous ones, but they cannot solve the absolute selective value of both types of substitutions. Also, the strong selective co-adaptation of bases on 1^st^, 2^nd^ and 3^rd^ codon positions (otherwise they cannot code) is overlooked and dealt with the necessary constraint of the genetic code. The present current position on acquisitions of pre-transcriptional evolution is to take them as un-debatable constraints (negative heuristic protective belt). For example, a replacement of adenine by guanine could change the velocity of DNA replication, leading to a great selective process that is not only invisible, but may be contradictory to codon analyses.

The foundations of the Neutral Theory were established after the discovery of the high frequency of polymorphisms that could not be maintained by balanced selection (heterozygous advantage; Kimura, 1968, 1979; Jukes and King, 1969; Nei, 2005). However, at the molecular level, the present genome studies show that for each polymorphic nucleotide site there are hundreds of monomorphic sites, so maintained for hundreds of millions of generations; such fixations can only be possible by selective evolution (Valenzuela and Santos, 1996; Valenzuela, 1997, 2000, 2002b, 2007).

The most important factual feature of evolution is the maintenance of genome sequences (the core to be a living being) for thousands of millions of cell cycle generations (think about unicellular and haploid organisms) through different taxa, and not polymorphisms or genetic variability. This is, perhaps, the most important restriction of the present evolutionary studies based on comparative phylogenetic analyses, which need genome variability among taxa and are blind for the evolution of the invariant part of genomes that is their largest proportion. The trans-taxa genome maintenance (fixation) contrasts with the individual genome instability. Individual genomes cannot be maintained during their ephemeral life (DNA mutations, cancer, aging). Post-transcriptional neutral-selective analyses cannot be performed on the major part of eukaryote genomes with more than 95% of non-coding sequences. Furthermore, these studies cannot quantify the effect of selection and drift on current genomes, the only approach to answer the question on the amount of neutral, nearly-neutral, selective and eventually pan-selective evolution. Foundational errors of the neutral theory do not allow solving these mentioned insufficiencies (Valenzuela and Santos, 1996; Valenzuela, 1997, 2000, 2002b, 2007). The random condition of neutral evolution implies reversibility, that is the transformation of unicellular organisms into multicellular ones should be as probable as the reverse process (Valenzuela, 2007). No study has shown a similar situation. Evolution is directional; we see convergence, not reversibility. The question is, how distant are genome sequences from randomness? Neutral evolution is incompatible with non-random distribution of nucleotides, but a random distribution of nucleotides is compatible with selective evolution.

Selective and neutral evolution imply mutation as the origin of variability. While neutral evolution proposes that drift plays a fundamental role in the population destiny of mutations and selection rarely contributes to the process, selective evolution proposes selection as fundamental and drift as a marginal or rare evolutionary process. A quantitative definition of rare or marginal has never been proposed, so as to be tested scientifically.

A methodology based on the quantitative and qualitative deviations of nucleotide sequences from the random neutral expected distribution was developed. This methodology is independent of post-transcriptional processes, phylogenetic variability and comparative analyses, but it includes their molecular bases. It detects selective processes where codon analyses do not show them and in genomes or genome segments that vary or do not vary among taxa. These analyses are complementary to coding and non-coding-region analyses or comparative studies to understand some selective mechanisms. Our first discovery (Valenzuela, 1985; Valenzuela and López-Fenner, 1986) was that the nucleotides' distribution on chromosomes follows a Bose-Einstein distribution of undistinguishable balls (nucleotides) on distinguishable boxes (chromosomes). Then, the expected neutral (random) chromosome length and centromere position could be calculated, founding the mathematical basis for chromosome evolution (Valenzuela, 1985; Gouet and López-Fenner, 1985, 1986; Valenzuela and López-Fenner, 1986).

The aim of the present study is to screen the whole genome of the HIV-1 virus and a segment that specify the envelope (GP120 *ENV*, S-env hereafter), and to establish and quantify their deviations from a random (neutral) distribution, without (intentionally) any reference to transcriptional processes or phylogeny. This approach was preceded by Gatlin (1976) who used the information theory to estimate the expected random sequence of coding DNA to test neutralism. She found a great deviation from randomness in DNA segments. Neutralists (Jukes, 1976; Kimura and Ohta, 1977) fast contra-argued that significant non-random sequence does not necessarily refute neutralism, because the mutation rate, in a site, could be influenced (a property of DNA or RNA polymerases) by the neighbor base context of this site. It was an undemonstrated negative heuristic protective hypothesis (an assumed neutral constraint that cannot be tested) to support the Neutral Theory. The debate closed without solution.

The position of Gatlin was considered satisfied by the unfounded neighbor influence, and non-random sequences were so accepted. However, neutralists did not realize that the neighbor influence does not change the expected random distribution of bases' sequences, because as a permanent property of polymerases, the neutral neighbor influence should also be isotropically and randomly distributed. Due to recurrent mutation, bases at any site are continuously changing; if evolution and the neighbor influence are neutral, the expected base at a site is a vector where the four bases are represented by a probability that is equal for all the sites (isotropy). In a short period, we should expect that each base has a proper neighborhood distributed isotropically along the genome, and the expected base, dinucleotide, trinucleotide, or any nucleotide sequence composition of long segments, should be equal, independently of its genome location.

Dividing genomes into long segments and comparing their mono or dinucleotide composition should test this isotropy of neutral evolution. Studies of base sequences have been performed and a great heterogeneity has been found. Significant different isochores (genome segments with similar base composition), maintained along with thousands of millions of generations, were found on every genome (Bernardi, 1993). These are macro-isochores (million bps), but micro-isochores (hundred or thousand bps) have also been found in fungi, bacteria and eukaryote organisms, both in coding and non-coding regions (Valenzuela, 1997; this article). Also big genome segments with different signatures (di-, tri- or multi-nucleotide structures) seem to be the rule in genomes (Karlin and Mrazek, 1997; Mrazek and Karlin, 2007; this article).

Besides isochores and signatures, a great deal of highly or moderately, tandemly repetitive DNA (VNTR, STR) or dispersed (LINEs, SINEs) in eukaryotes show high intra and inter chromosome correlations. The acquisition and maintenance of isochores, signatures and repetitive DNA for hundreds of millions of generations, and their wide intra and inter-chromosome variability refute definitively neutral and nearly neutral evolution and the neutral neighbor influence. It astonishes how the scientific community seems blind or unaware of this conclusive refutation. The random motion of the sand (bases) may build a sand castle (genome), but it cannot maintain the castle, on the contrary, it is the main cause of its destruction (Valenzuela, 2007).

It is important to note that the neighbor influence hypothesis is also valid for selective evolution, because a DNA or RNA sequence could have higher adaptive values than other sequences, as it will be shown in this article. So, the neighbor influence hypothesis rather blurs than helps to solve the selective-neutral condition of evolution. In the present study, conclusive evidence is given on the existence of micro-isochores and micro-signatures among the HIV-1 and S-env base sequences. HIV-1 was chosen because viruses evolve fast (Drake, 1993, 1999; Drake *et al.*, 1998). There are different lines of evidence showing that S-env is under selective pressure (Reiher *et al.*, 1986; Serres, 2001; Yang, 2001; Mani *et al.*, 2002; Kitrinos *et al.*, 2003; Travers *et al.*, 2005; MacNeil *et al.*, 2007). On the other hand, neutral molecular evolution has also been proposed for this gene (Leigh-Brown, 1997; Zhang, 2004).

Here I propose a method to estimate the distance from randomness (neutralism) for any DNA, RNA or amino-acid sequence, independently of the taxon at which it belongs, to test how much distant are genomes or genome segments (the core of living beings) from random processes. This method allows measurements of the distance from randomness of that part of living beings by which they stand as living beings (Valenzuela, 2002a).

## Material and Methods

Complete cDNA sequence of the HIV-1 virus was obtained from Genbank (accession number AF005495, isolated in Brazil). Also, a cDNA sequence of the GP120 *ENV* gene (S-env) of the HIV-1 was used (accession number AF119820, from Cyprus and Greece). Abbreviations A, T, G and C will be used for Adenine, Thymine, Guanine and Cytosine; their base frequencies will be denoted by f_A_, f_T_, f_G_ and f_C_, and their number by N_A_, N_T_, N_G_ and N_C_, respectively. Degrees of freedom (DF) for tests are subscript. For huge values of the χ^2^_k_ test (k DF) an approximation was made taking into account that χ^2^_k_ distribution has mean k and variance 2k, then, an extrapolation may be obtained for the decay of the probability according to the number of standard deviations from the χ^2^ value and the mean (as a z test, with a correction made by the deviation of the χ^2^ from the Gaussian distribution according to DF, using known data of the χ^2^ distribution). For small-expected numbers (< 5) the Poisson distribution was used to calculate significance. For large values of z, the proposition of Freund *et al.*, (2000) for one-tailed test was used: Probability for 4z = 0.49997; 5z = 0.4999997; 6z = 0.499999999; and extrapolation, according to this tendency, for larger z.

### Rationale

Under neutral evolution, mutation and drift are the main evolutionary factors; the probability to find any of the four bases at any nucleotide site is the same. This probability has been shown to be 0.25 for the four bases, accepting equal mutation rates among them (Jukes and Cantor, 1969; Valenzuela and Santos, 1996; Li 1997). If transitions and transversions occur with different mutation rates, the expected base frequency will still be 0.25 for the four bases (Valenzuela and Santos, 1996). These probabilities change with different mutation rates among the bases, however due to the complementariness of A-T, and G-C, six parameters are sufficient to describe the system (Sueoka, 1995; Valenzuela, 1997); in this condition the expected f_A_ equates f_T_ and the same occurs with f_G_ and f_C_. These equalities are not expected for single stranded nucleic acid where complementariness is not possible.

### Analysis of the expected equal proportions of A-T and G-C

If expected f_A_ = expected f_T_ and f_G_ = f_C_, then N_A_ = N_T_ and N_G_ = N_C_. Both equalities can be tested by a χ^2^_1_ test for equality where the expected number are EN_A-T_ = (N_A_ + N_T_)/2 and EN_G-C_ = (N_G_ + N_C_)/2, respectively. Thus, χ^2^_1_,_A-T_ = 2x(N_A_ - EN_A-T_)^2^/EN_A-T_ and χ^2^_1_,_G-C_ = 2x(N_G_ - EN_G-C_)^2^/EN_G-C_.

### Analyses of the neutral expected homogeneity of di- and mononucleotide proportion

The influence of a base on mutation rates of neighbor sites does not change the equal expectancy of the four bases in a site, because the historical average influence of the neighbor bases in a site is the same for every site. If neighbor influence is true, it is expected that in short historical periods a base will be associated with a particular vector frequency of the four bases in the neighbor sites along with the whole genome. For neutral evolution, this frequency vector should be stochastically invariant along the genome, and this can be tested by examining the homogeneity of bases or dinucleotides in sufficiently long sub-segments of a DNA (RNA) segment. If this influence is neutral, in evolutionary periods (millions of generations or more), it should be balanced by the turnover of the four bases in this site. Here, “long” depends on the extension of the influence, thus, for our purpose, it is more than 10 sites, because we found that this influence for DNA genes is highly significant in consecutive bases (0 site separation), it decays greatly for bases separated by one site, two sites and it is not significant for separations equal or longer than three sites. This occurs in DNA segments; RNA viruses are expected to have a wider neighborhood, because RNA should be processed and folded to be put into the envelope (capsid), requiring that any site correlate with any other.

### Analyses of base and no-base sequences

A base, for example A, may be consecutively present 0, 1(A), 2(AA), 3(AAA), n times in a DNA segment (Supplementary Material, S1). In the same segment “No-A” (Z = T, G and C) may be present 0, 1(Z), 2(ZZ), 3(ZZZ), n times. The set of A, with N_A_ bases in a DNA segment may be taken as a set of undistinguishable balls and the set of Z, with N_Z_+1 No-A bases, may be taken as the walls of distinguishable boxes where balls are distributed, and vice-versa (with N_Z_ balls and N_A_+1 boxes). The random distribution of undistinguishable balls in distinguishable boxes follows a Bose-Einstein (B-E) statistics (Feller, 1968; Supplementary Material, S1). With this expected random (neutral) distribution, the observed distribution of bases and no-bases was tested; total comparison is obtained by a χ^2^_k-1_, k being the number of non-0 cases of numbers of balls in a box. We can also test the observed variance with the expected B-E random variance with a specific test developed for this purpose (Supplementary Material, S1); this is the analysis of the variance of the variance. The number of a base and no-base runs can be tested with the non-parametric run-test (Supplementary Material, Appendix S2; Freund *et al.*, 2000; Spiegel *et al.*, 2001). These three analyses were applied to the total HIV-1 and to S-env DNA segment. The three tests are based on the B-E distribution, their information overlaps partly, but they also inform on independent traits of deviations from randomness. The analyses of the number of consecutive bases and no-bases inform on the general and specific distribution of a base and no-base; the analysis of the variance of the variance informs on how much clustered or widespread are the sequences of bases or no-bases (uni, bi or multiple modality); the run analysis informs on the tendency of bases and no-bases to cluster in series or to be isolated. Base sequences can be also analyzed with the Geometric distribution, assuming p as the probability to find a base and q = (1-p) the probability of finding a no-base (Valenzuela and Santos, 1996). Here only B-E analyses are performed.

## Results and Discussions

The number of nucleotide sites for the whole genome of HIV-1 was 8954; N_A_ = 3236 (36.14%); N_T_ = 1964 (21.93%); N_G_ = 2173 (24.27%); N_C_ = 1581 (17.66%). The number of sites for S-env was 2627; N_A_ = 901 (34.30%); N_T_ = 636 (24.21%); N_G_ = 621 (23.64%); N_C_ = 469 (17.85%). The difference in base composition of both DNA segments was near the significance level (χ^2^_3_ = 6.99, p = 0.072); this figure should be considered significant because positive covariance between S-env and HIV-1 base composition was not considered. The isolated f_T_ was significantly higher in S-env (z for proportion = 2.48, p = 0.013). Both base compositions are significantly different from the expected neutral distribution of 0.25 for each base (no test is needed).

### Tests for equal numbers of complementary bases

**HIV-1**: χ^2^_1_,_A-T_ = 311.15, p < 10^-60^; χ^2^_1,G-C_ = 93.35, p < 10^-19^.

**S-env**: χ^2^_1,A-T_ = 45.69, p < 10^-9^; χ^2^_1,G-C_ = 21.20, p = 4.2 x 10^-6^.

Figures are different from the expected A-T and G-C equalities; this may be due to the fact that this is a single stranded RNA retrovirus, but an important part of its cycle occurs as DNA, in the host genome.

### Homogeneity tests for proportions and distribution of di- and mono-nucleotides (bases)

**HIV-1**. Table 1 shows the random-Expected and Observed distribution of overlapping dinucleotides of HIV-1 separated by 0, 1, 2 and 3 nucleotide sites. The χ^2^_9_ values decayed strongly from consecutive (0 separation) dinucleotides (p < 10^-80^) to those separated by 1 (p < 10^-30^), 2 (p = 0.000016) and 3 (p = 0.01736) nucleotide sites. An important part of significance found in 1 and 2 sites separation matrices may be due to the big deviation present in consecutive sites. This indicates that the neutral neighbor influence, if real, is mostly reduced to one or, at most and slightly, to two sites.

The study was carried out with separations until 33 sites, finding significant values that ranged between 1 and 5% for separations over three sites, with a few exceptions, as that observed for 8 separation sites (p = 0.401). This is a mystery, because mononucleotide pairs with 2, 4, and 16 separation sites (which include those with 8 sites away) were significantly correlated (deviated from randomness); with 32 separation sites, no deviation from randomness was found (p = 0.557), but that deviation was observed for 31 and 33 (p = 0.009, p = 0.0000046). The study of waves of correlations among sites is out of the scope of this article. This agrees with our intuitive prediction for single stranded RNA segments that could be packed into a capsid. All these correlations cannot be due to random influences and refute neutralism, indicating that a nucleotide at any site must correlate with the whole context of a small single-stranded RNA genome to be maintained. Our analyses on eukaryote genomes show a different picture, where correlations of this type are restricted to one or at most two sites; separations of more than 2 sites yield non-significant values (unpublished results).

The structure of dinucleotides (0 site separation) showed significant deviations from randomness, ranging from more to less significant as follows: lack of CG, excess of CA, excess of AG, excess of GG, excess of CC, lack of GT, excess of TT, excess of CT, lack of TC, and lack of GA. The lack of CG is found widespread in eukaryote genomes that inactivate genes by means of methylation of C in CpG dinucleotides (often promoters). However, this is a RNA virus that can be incorporated to the host genome.

It is straightforward to propose that, either it is a selective adaptation to host CpG inactivation mechanisms of RNA viral genome, or HIV-1 or its ancestors were incorporated in the primate genome several million years ago and shares with hosts the same inactivation mechanism. In both cases, this is a strong evidence for selective adaptation; it is still possible to invocate the neutral neighbor influence, but the level of significance (Expected 383.7, observed 79, χ^2^_1_ = 241.99, p < 10^-50^) makes this mechanism untenable.

HIV-1 appeared in humans not more than 70 years ago, an insufficient time to produce such a deviation from the expected neutral random distribution, moreover, due to its high mutation rate (Drake, 1993, 1999; Drake *et al.*, 1998), and if evolution is (mutations are) mostly neutral, this is a sufficient time to yield a near random neutral base distribution. Thus, the hypothesis that this dinucleotide structure appeared in primates several millions of generations ago and is maintained by selection until the present human infection is strongly affirmed. Moreover, the observed number of the symmetric (main diagonal) GC pair, that theoretically must have the same frequency as CG (if evolution is neutral), was 421, not significantly different from the expected number (383.5). Thus, to maintain the neutral theory, it is necessary, besides the addition of a very especial kind of neighbor influence, the addition of the hypothesis of polarity (5'-3') discrimination of both CG and GC pairs for mutation and neighbor influence. Five of the six symmetrical dinucleotides showed significant differences [941(AG) *vs.* 727(GA); 607(AC) *vs.* 739(CA); 507(TG) *vs.* 379(GT); 293(TC) *vs.* 404(CT); and the 421(GC) *vs.* 79(CG) already shown]. The existence of a very similar virus in chimpanzees is a well-known fact (Jern *et al.*, 2006) corroborating these results, but, inferences of the present study do not need phylogenetic information and are founded only on its analyses and deduction from theoretical background. It is impressive that the dinucleotide structure found with 0 site separation (0SS) disappears and is reversed with one site separation (1SS). The case of the highly lack (p < 10^-50^) of CG in 0SS reverted to a significant excess (p = 0.0015) in 1SS is dramatic.

Let us assume (better imagine) that neutral evolution, with the addition of the neighbor influence and the 3'-5' discrimination has produced and maintained these huge deviations from randomness (even though this is factually impossible). There is still an independent test for neutralism, because these deviations should be distributed homogeneously along with the whole HIV-1 genome. Table 2 presents the division of HIV-1 genome in 10 equal sub-segments and the analyses for di- and mononucleotide distributions. The huge heterogeneity of dinucleotide (p < 10^-20^) and mononucleotide (p < 10^-15^) distributions refutes definitively neutral evolution and the neighbor influence.

Let us examine a case, in dinucleotides, the mononucleotide frequency vector associated to A (first four rows) in segment 4° is (f_A_ = 0.3924; f_T_ = 0.1907; f_G_ = 0.2643; f_C_ = 0.1526) and in segment 10° is (f_A_ = 0.2784; f_T_ = 0.1412; f_G_ = 0.3725; f_C_ = 0.2078). There is no known property of polymerases that enables them to distinguish A of the segment 4° from A of the segment 10°, so as to yield such different mutation rates leading to these different vectors of the contiguous nucleotide. The heterogeneities of the nucleotide frequency vector, in the 10 sub-segments, associated to A, T, G, and C were: χ^2^_27_ = 56.9, p = 0.00066; χ^2^_27_ = 41.0, p = 0.0412; χ^2^_27_ = 49.2, p = 0.0056; and χ^2^_27_ = 90.9, p = 0.0000015, respectively. The same high heterogeneity occurs among nucleotide frequencies. It is impossible for neutral mutation rates, genetic drift and the neighbor influence to produce and maintain such deviations from the expected random distribution.

**S-env**. Table 3 shows the dinucleotide distribution of S-env with 0, 1, 2 and 3 separation sites. The distribution is similar to that of HIV-1 whole genome. Significances are smaller due to smaller numbers. The same similarity of both segments is found in Table 4 that presents di- and mononucleotides in three equal sub-segments (to work with S-env sub-segments similar to HIV-1 sub-segments) of S-env sequence. Even though S-env has near 25% of the total HIV-1 genome and a significant deviation from randomness of the mononucleotide distribution in the three sub-segments was expected, data agreed with randomness instead. Also the variance of the dinucleotide composition was higher (not significantly) in HIV-1 than in S-env [see percents in the last column of Tables 2 (from 12.4 to 0.9) and 4 (from 11.1 to 1.2), respectively]. This is a very interesting result that we have found consistently.

DNA segments submitted to known higher pressures of selection, as for example coding regions, are not necessarily more deviated from randomness (in nucleotide sequences) than less selective segments (non-coding regions). This is expected due to the constraint of codons (triplets) in coding regions or to selective constraints that do not allow for a great variability of nucleotide sequences. Non-coding regions can accept a long repeat of mono-, di-, tri-, tetra- or multi-nucleotides that coding regions cannot. Evolutionary studies of post-transcriptional processes are blind for evolution of pre-transcriptional ones that do not have a consequence on coding variability. Furthermore, studies on variable regions of genomes (polymorphism) are blind for selective processes of non-polymorphic regions that are by far more frequent than variables ones. As we indicated, the most important evolutionary problem is not variability or the maintenance of variability, but invariance or the maintenance of invariance along with millions of generations. The maintenance (fixation) of similarities (invariants) is impossible for neutral or nearly neutral evolution (Valenzuela and Santos, 1996; Valenzuela, 2000, 2007). As it was remarked our individual genome is unstable, we die inexorably by mutation (cancer and aging), but the *Homo sapiens* genome is more stable than the individual one due to selection within the species and higher taxa.

### Analyses of sequences of isolated bases or no-bases (Bose-Einstein analyses)

**HIV-1**. Table 5 presents this analysis for the HIV-1 complete genome. The statistical significance of isolated number of runs of bases is superscript. **Adenine, A**: only an excess of 1A was significant (p = 0.0019), however, the total distribution was significantly different from randomness (p = 0.00013), thus A showed no tendency to cluster and an observed variance of A distribution (A-OVar) smaller but not significantly different from the expected value (z = 0.94, p = 0.3472); both results indicate that A is more dispersed than expected. **No-A**: a significant excess of 1No-A (p = 0.006), 2No-A (p = 0.012), 9No-A (p = 0.012) and 23No-A (p = 0.038) were found, thus No-A showed a slight tendency to be both isolated and cluster in couples and 23No-A tandem, the total distribution being significantly deviated from randomness (p = 0.0005), no significant higher No-A-OVar than expected difference was found (z = 1.4, p = 0.1585). The runs of A and No-A yielded z = 2.8, p = 0.0045, the positive value indicates that there were more runs than the expected mean, confirming that A and No-A are more dispersed than expected from a random B-E distribution.

**Thymine**: the frequency of 1T was significantly less than expected (p = 0.0054), an excess was found for 4T (p = 0.018), giving a significant total (p = 0.0014), thus T showed a mild tendency to cluster; the observed T-OVar was significantly greater than expected (z = 2.83, p = 0.0047). **No-T**: as expected from the T distribution, the category 0No-T showed a significant excess (p = 0.00075), because T showed a tendency to be clustered; other excesses were found in 22No-T (p = 0.00995), 39No-T (p = 0.027) and 40No-T (p = 0.022); 3No-T presented a slight loss (p = 0.03); the total was also significant (p = 0.00017), in favor of clusters of No-T; No-T-OVar was larger than expected (z = 3.5, p = 0.00045). The run test for T and No-T yielded z = -4.3, p = 0.000015, indicating less runs than randomly expected; this confirms the tendency of T-No-T to cluster.

**Guanine:** G showed less 1G than expected (p = 2.9 x 10^-6^) and excesses of 4G (p = 0.00032) and 6G (p = 0.0001), being the total deviation from randomness highly significant (p = 6 x 10^-8^); G-OVar was significantly larger than expected (z = 4.4, p = 0.00001), thus, G showed a strong tendency to cluster. **No-G**: 0No-G was more frequent than expected (p < 10^-8^) and 1No-G less than expected (p < 10^-10^), there was an excess of 36No-G (p = 0.023); the total deviation was also highly significant (p < 10^-8^); the tendency to cluster was not so marked as for G; No-G-OVar was greater than expected, but close to significant values (p = 0.074). There were less G and No-G runs than the expected mean (z = -6.8, p < 10^-8^), confirming the tendency of G-No-G to cluster.

**Cytosine:** a significant deficiency of 1C (p = 0.00007), and excesses of 3C (p = 0.00036) and 4C (p = 0.0358) were the features of C distribution, that showed a clear tendency to cluster (in 3 and 4 C); the total deviation from randomness was significant (p = 1.4 x 10^-6^); C-OVar was larger than expected (z = 2.9, p = 0.0032). **No-C:** 0No-C, 31No-C and 42No-C showed an excess (p = 1.9 x 10^-6^, p = 0.034, p = 0.004, respectively), No-C and 3No-C showed a deficiency (p = 0.0027 and 0.0379, respectively), thus the tendency to cluster was evident; the total deviation was significant (p = 10^-8^); No-C-OVar was larger than expected (z = 2.31, p = 0.0209). There were less C and No-C runs than expected (z = -5.8, p < 10^-8^), verifying the tendency of C and No-C to cluster.

**S-env.** It is important to remark that this S-env came from another HIV-1 strain than the HIV-1 whole genome. However, the general structure of deviations from randomness of sequences of bases and No-bases was similar to that of the complete HIV-1, as expected, with less significant figures due to the smaller number of nucleotide sites. A few disagreements between env and HIV-1 values should be remarked. Adenine: in S-env there were more 2A dinucleotides than expected (p = 0.0028); in HIV-1 there were less. Thymine: S-env showed more 1T than expected (non-significant); HIV-1 had less observed than expected 1T (p = 0.007). No-T: A highly excess of 0No-T (p < 0.001), in HIV-1, was not correlated with a small deficiency (p = 0.8) in S-env. A significant excess of 2No-T (p = 0.013), in S-env, was not found in HIV-1, which instead presented a non-significant deficiency of 2No-T. G, No-G, C and No-C did not show differences in both segments. The results of analyses of OVar and runs were consistent with those found in HIV-1. This last agreement between HIV-1 whole genome and S-env, on addition to similar distribution of dinucleotides, allows assigning S-env to HIV-1 (or to a similar retro-virus) with high confidence, even ignoring its real origin.

## Conclusions

(1) Neutral mutations and random drift cannot produce and maintain the huge deviations from randomness found in the base sequences of HIV-1 and S-env, huge deviations from randomness and significant mono- and di-nucleotide heterogeneities are present in segments of less than 1000 bp. (2) There is a significant dinucleotide correlation (non-random distribution) among all the sites of the whole HIV-1 virus. (3) The high heterogeneity of sub-segments of HIV-1 or S-env sub-segments refutes conclusively the neutral neighbor influence. (4) The dinucleotide structure (signature) of HIV-1 and S-env show some traits of eukaryote signatures, not expected for a RNA virus, suggesting that HIV-1 virus has co-evolved within ape genomes for millions of gamete and viral generations. (5) These findings suggest that pre-transcriptional evolution of HIV-1, with its pre-human stage, is pan-selective rather than neutral or nearly neutral.

A metaphor may give a better understanding of conclusions. If bases are words of a language, neutral and nearly neutral evolution should yield an average random neighbor around any word, aperiodically repeated throughout the whole tale; they should write similar stochastic tales, collected in a stochastic library; the present study shows that any segment has a meaningful sequence distant from randomness, never exactly repeated and with a high correlation among all the words of the tale; they should write different meaningful adaptive tales, collected in the library of life.

## Figures and Tables

**Table 1:** Random expected and observed overlapping di-nucleotides of HIV-1, with separation of 0, 1, 2 and 3 sites.

1° Base		2° Base						2° Base				
		0 site separation						1 site separation				
		A	T	G	C	Tot		A	T	G	C	Tot
A	O E	1107 1169.3	680 709.7	941 785.2	607 570.9	3235		1233 1169.4	738 709.4	703 785.3	561 571.0	3235
T	O E	663 709.9	501 430.8	507 476.7	293 346.6	1964		601 709.6	472 430.5	529 476.5	361 346.5	1963
G	O E	727 785.4	379 476.7	646 527.4	421 383.5	2173		887 785.5	395 476.5	495 527.5	396 383.5	2173
C	O E	739 571.4	404 346.8	79 383.7	359 279.0	1581		515 571.5	358 346.7	446 383.8	262 279.0	1589
Tot		3236	1964	2173	1580	8953		3236	1963	2173	1580	8952

		χ^2^_9_ = 445.55, p < 10^-80^						χ^2^_9_ = 86.98, p < 10^-30^				

		2 sites' separation						3 sites' separation				

A	O E	1256 1169.5	697 709.5	750 785.0	522 571.0	3235		1190 1169.3	672 709.5	791 785.1	582 571.1	3235
T	O E	688 709.3	458 430.3	458 476.1	358 346.3	1962		738 709.2	426 430.3	479 476.1	319 346.4	1962
G	O E	789 785.6	430 476.6	568 527.3	386 383.6	2173		719 785.4	520 476.6	527 527.4	412 383.6	2173
C	O E	503 571.6	378 346.7	386 383.6	314 279.1	1581		593 571.1	345 346.5	375 383.4	267 278.9	1580
Tot		3236	1963	2172	1580	8951		3235	1963	2172	1580	8950

		χ^2^_9_ = 38.28, p = 0.000016						χ^2^_9_ = 20.09, p = 0.01736				

O = observed; E = expected; Tot = total; p = probability.

**Table 2:** Di- and mono-nucleotides on 10 segments of the HIV-1.

	Dinucleotides of segments 1° TO 10°(χ^2^_135_ = 327.6; p < 10^-20^)											
Pair	1°	2°	3°	4°	5°	6°	7°	8°	9°	10°	Total	
											N	%
AA	114	119	127	144	125	88	107	126	86	71	1107	12.4
AT	58	63	79	70	72	70	90	80	61	36	679	7.6
AG	109	99	82	97	106	103	78	81	89	95	939	10.5
AC	55	46	56	56	49	49	47	53	41	53	505	5.6
TA	66	52	70	71	73	65	91	70	54	49	661	7.4
TT	34	56	61	41	48	60	49	45	52	53	499	5.6
TG	36	43	52	42	42	46	66	53	65	62	507	5.7
TC	21	28	32	25	24	35	26	32	37	33	293	3.3
GA	78	83	72	73	70	76	53	67	80	75	727	8.1
GT	29	27	40	38	44	33	54	43	36	35	379	4.2
GG	69	70	48	59	66	61	47	58	77	90	645	7.2
GC	55	40	24	32	39	44	40	37	53	55	419	4.7
CA	79	74	75	79	84	81	71	78	58	60	739	8.3
CT	36	33	35	31	23	42	40	31	59	73	403	4.5
CG	17	7	3	3	5	5	3	13	15	8	79	0.9
CC	38	54	38	33	24	36	32	27	31	46	359	4.0
Tot	894	894	894	894	894	894	894	894	894	894	8940	100.0

Mononucleotides of segments 1° TO 10°(χ^22^_7_ = 87.1; p < 10^-15^)												
A	337	328	344	367	352	310	322	341	278	255	3234	36.1
T	157	179	215	180	187	206	233	200	208	197	1962	21.9
G	231	220	185	202	219	215	194	205	246	255	2172	24.3
C	170	168	151	146	136	164	146	149	163	187	1580	17.7
Tot	895	895	895	895	894	895	895	895	895	894	8948	100.0

**Table 3:** Random expected and observed overlapping di-nucleotides of S-env, with separation of 0, 1, 2 and 3 sites.

1° Base		2° Base						2° Base				
		0 site separation						1 site separation				
		A	T	G	C	Tot		A	T	G	C	Tot
A	O E	292 308.9	220 218.1	224 212.9	164 160.8	900		314 308.8	242 218.0	179 212.8	164 160.7	899
T	O E	222 218.1	151 153.9	184 150.3	79 113.5	636		202 218.0	162 153.9	160 150.2	112 113.5	636
G	O E	191 212.9	123 150.3	182 146.7	125 110.8	621		243 212.8	124 150.2	160 146.7	94 110.8	621
C	O E	195 160.8	142 113.5	31 110.8	101 83.7	469		141 160.7	107 113.5	122 110.8	94 83.7	469
Tot		900	636	621	469	2626		900	635	621	469	2625

		χ^2^_9_ = 112.8, p < 10^-15^						χ^2^_9_ = 28.3, p = 0.00085				

		2 sites' separation						3 sites' separation				

A	O E	350 308.7	200 217.9	202 212.7	147 160.7	899		305 308.6	213 217.8	226 212.7	155 160.6	899
T	O E	207 217.9	173 153.8	151 150.2	104 113.4	635		234 217.8	137 153.7	163 150.1	100 113.4	634
G	O E	205 212.7	135 150.2	157 146.6	124 110.7	621		201 212.7	166 150.1	126 146.6	128 110.7	621
C	O E	138 160.7	127 113.4	110 110.7	94 83.6	469		159 160.6	119 113.4	105 110.7	86 83.6	469
Tot		900	635	620	469	2624		899	635	620	469	2623

		χ^2^_9_ = 22.7, p = 0.0069						χ^2^_9_ = 15.4, p = 0.0805				

O = observed; E = expected; Tot = total; p = probability.

**Table 4:** Di- and mono-nucleotides of three segments of S-env.

Dinucleotides of segments 1° TO 3° (χ^2^_30_ = 67.66; p = 0.000098)					
Pair	1°	2°	3°	Total	
				N	%
AA	104	98	89	291	11.1
AT	84	72	64	220	8.4
AG	66	85	73	224	8.5
AC	51	66	47	164	6.3
TA	82	77	62	221	8.4
TT	48	41	61	150	5.7
TG	72	50	61	183	7.0
TC	23	22	34	79	3.0
GA	53	67	71	191	7.3
GT	54	42	27	123	4.7
GG	48	64	70	182	6.9
GC	37	38	50	125	4.8
CA	65	79	51	195	7.4
CT	40	35	67	142	5.4
CG	6	12	13	31	1.2
CC	41	26	34	101	3.9
Tot	874	874	874	2622	100.0

Mononucleotides of segments 1° TO 3° (χ^2^_6_ = 9.899; p = 0.1290)					
A	305	321	273	899	34.3
T	226	190	219	635	24.2
G	192	211	218	621	23.7
C	152	152	165	469	17.8
Tot	875	874	875	2624	100.0

**Table 5:**
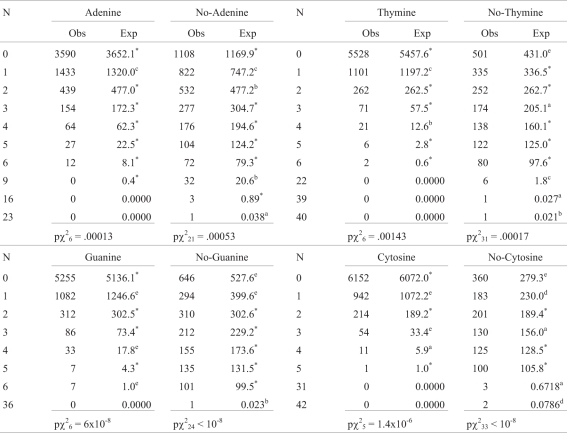
Observed and expected numbers of base and no-base runs.

N = number of consecutive bases in a run; p = probability; ^*^= p ≥ 0.05; ^a^= 0.05 > p ≥ 0.025; ^b^= 0.25 > p ≥ 0.01; ^c^= .01 > p ≥ 0.005; ^d^= 0.005 > p ≥ 0.001; ^e^ = p < 0.001.

## Supplementary Material

The following online material is available for this article:

Appendix S1 Bose-Einstein (B-E) distribution of nucleotides in a DNA segment.

Appendix S2 Testing Runs.

This material is available as part of the online article from http://www.scielo.br/gmb.
