# Supplementary material for: Non-random pre-transcriptional evolution in HIV-1. A refutation of the foundational conditions for neutral evolution
Source: Genet Mol Biol. 2009 Jan 30;32(1):159–69. doi: 10.1590/S1415-47572009005000025 (PMC3032973; doi:10.1590/S1415-47572009005000025)
Supplement: Appendix S1 — Bose-Einstein (B-E) distribution of nucleotides in a DNA segment. [file gmb-32-1-159-suppl1.pdf]

Supplementary Material

S1. Bose-Einstein (B-E) distribution of nucleotides in a DNA segment

A DNA segment with 3 A (N_A=3=R) and 4 No-A (Z = T, G, C) bases (N_Z=4=N-1) may have a sequence AZZAZAZ. Zs may be walls of boxes where balls A can be put in. We add one wall to N_Z to obtain the number of boxes (R balls in N boxes). The number of different arrays of R indistinguishable As in N distinguishable boxes is (N+R-1)!/[R!(N-1)!]; these arrays are distributed with a Bose-Einstein (B-E) statistics (Feller, 1968). These are combinations of N_A+N_Z bases taken N_A or N_Z at a time. Let C(N,R) be the number of arrays of R balls (N_A) in N boxes $(N\text{-}1)_Z$; C(5,3)=7!/(3!4!)=35 different arrays, in the example:

<table>
<tr><th></th><th colspan="2">0 A in the first box</th><th>1 A in the first box</th><th>2 As in the first box</th></tr>
<tr><td>1</td><td>|ZZZZAAA|</td><td>|ZAZZZAA|</td><td>|AZZZZAA|</td><td>|AAZZZZA|</td></tr>
<tr><td>2</td><td>|ZZZAZAA|</td><td>|ZAAZZZA|</td><td>|AZZZAZA|</td><td>|AAZZZAZ|</td></tr>
<tr><td>3</td><td>|ZZZAAZA|</td><td>|ZAAAZZZ|</td><td>|AZZZAAZ|</td><td>|AAZAZZZ|</td></tr>
<tr><td>4</td><td>|ZZZAAAZ|</td><td>|ZAZZAZA|</td><td>|AZZAZZA|</td><td>|AAZZAZZ|</td></tr>
<tr><td>5</td><td>|ZZAZZAA|</td><td>|ZAZZAAZ|</td><td>|AZZAAZZ|</td><td></td></tr>
<tr><td>6</td><td>|ZZAAZZA|</td><td>|ZAAZZAZ|</td><td>|AZZAZAZ|</td><td></td></tr>
<tr><td>7</td><td>|ZZAAAZZ|</td><td>|ZAZAZZA|</td><td>|AZAZZZA|</td><td></td></tr>
<tr><td>8</td><td>|ZZAZAAZ|</td><td>|ZAAZAZZ|</td><td>|AZAAZZZ|</td><td>3 As in the</td></tr>
<tr><td>9</td><td>|ZZAAZAZ|</td><td>|ZAZAAZZ|</td><td>|AZAZZAZ|</td><td>first box</td></tr>
<tr><td>10</td><td>|ZZAZAZA|</td><td>|ZAZAZAZ|</td><td>|AZAZAZZ|</td><td>|AAAZZZZ|</td></tr>
</table>

The expected mean of A per box (in the first box) is (20x0+10x1+ 4x2+1x3)/35=21/35=3/5; this occurs equally for each box; that is

N_A divided by N_Z+1. B-E distribution has the same coefficients of the Pascal's Triangle as in the following matrix: number of arrays given by R balls in N boxes {C(N,R) = (N+R-1)!/[R!(N-1)!]}, where the binomial coefficients are in the secondary diagonals.

	R						
N	0	1	2	3	4	5 …	R
1	1	1	1	1	1	1 …	R!/(R!0!)
2	1	2	3	4	5	6 …	(R+1)!/(R!1!)
3	1	3	6	10	15	21 …	(R+2)!/(R!2!)
4	1	4	10	20	35	56 …	(R+3)!/(R!3!)
5	1	5	15	35	70	126 …	(R+4)!/(R!4!)
6	1	6	21	56	126	252 …	(R+5)!/(R!5!)
7	1	7	28	84	210	462 …	(R+6)!/(R!6!)
8	1	8	36	120	330	792 …	(R+7)!/(R!7!)
…	1	…	…	…	…	…	…
N	$\frac{(N-1)!}{0!(N-1)!}$	$\frac{N!}{1!(N-1)!}$	$\frac{(N+1)!}{2!(N-1)!}$	$\frac{(N+2)!}{3!(N-1)!}$	$\frac{(N+3)!}{4!(N-1)!}$	$\frac{(N+4)!}{5!(N-1)!}$ …	$\frac{(N+R-1)!}{R!(N-1)}$

We demonstrate that any element in the matrix is the sum of the elements in the preceding row or column, that is C(N,R)=C[(N-1),R]+C[(N-1),(R-1)]+C[(N-1),(R-2)]+... C[(N-1),(R-R)]; or C(N,R)=C[N,(R-1)]+C[(N -1),(R-1)]+C[(N-2),(R-1)]+... C[(N-N+1),(R-1)] for rows or columns respectively. Let us demonstrate it for rows. If a box is fixed, as in the first box of the example, it can have 0, 1, 2, ..., R balls. This series describes exhaustively and without repetition the universe of arrays. If the box is empty (0 balls) the number of arrays is C[(N-1),R], the element of the matrix immediately over C(N,R),

because R balls distribute in (N-1) boxes. If the box has only one ball, the number of arrays is C[(N-1),(R-1)] because, R-1 balls remain to be distributed in (N-1) boxes. The series continues, from right to left, until all the balls are in this box in only one way. The demonstration for columns is similar, though, less evident; it can be done by symmetry with rows. Now, by mathematical induction we demonstrate that the formula holds for any element of the matrix. For the first row C(1,R) = 1, for all the elements in the series (1+R-1)!/(R!0!)=1. For the first column C(N,0)= 1, (N+0-1)!/[0!(N-1)!]=1. For the second row and column we have: C(2,R) = (2+R-1)!/[R!(2-1)!] = R+1, and C(N, 1) = (N+1-1)!/[1!(N -1)!] = N, respectively. Then we show that any element of the matrix is generated by the addition of the preceding element of the row and the preceding element of the column, that is: C(N,R)=C(N,R-1)+C(N-1,R), for

$$\frac{(N+R-1-1)!}{(R-1)!(N-1)!} + \frac{(N-1+R-1)!}{R!(N-2)!} = \frac{(N+R-2)!R + (N+R-2)!(N-1)}{R!(N-1)!} = \frac{(N+R-2)!(R+N-1)}{R!(N-1)!}$$

$$= \frac{(N+R-1)!}{R!(N-1)!} = C(N,R)$$

From this analysis, the expected probability of having 0, 1, 2,...R balls in a box is given by the development of the numerator of the quotient C(N-1,R)/C(N,R). They are (N+R-2)!R!(N -1)!/[(N+R-1)!R!(N-2)!]=(N-1)/(N+R-1); C(N-1,R-1)/C(N,R)=(N-1)R/ [(N+R-1)(N+R-2)]; C(N-1,R-2)/C(N,R)=(N-1)R(R-1)/[(N+R-1)(N+R-2)(N+R-3)];...C(N-1,R-R)/C(N,R)=(N-2)!/[(N-2)!0!]=1, respectively. These formulae allow to test observed numbers of series with 0, 1, 2, R nucleotides, which can be found when R nucleotides distribute in N boxes [$(N-1)_Z$]. In the example they are P(0)=20/

35=0.571; P(1)=10/35=0.286; P(2)=4/35=0.114 and P(3)=1/35=0.029. Expected numbers of boxes with 0, 1, 2...R balls are the product of these probabilities and the number of boxes (N), that is, in the example, N(0)=4x0.571=2.284; N(1)= 1.144; N(2)=0.456 and N(3)=0.116. Let us obtain the mean [E(x), x = number of balls in a box] and variance Var(x)={E(x^2)-$[E(x)]^2$} of this distribution. Elements of the B-E matrix are obtained by summing up precedent row or column elements. Thus, the sum of values (x), the sum of squares (x^2), higher order moments and their probability (division by the respective element which is the total number of arrays) are obtained by a linear combination of some elements of the matrix. Let us calculate the mean and variance of x corresponding to C(N,R)= ΣC(N-1,R-J); J (the value of x) goes from 0 to R (reading B-E matrix from right to left). Since C(N-1,R) is the number of arrays where x=0, which does not contribute to the sum of x or x^2, the calculus is made from R-1. The development of C(N+2,R-1), written from right to left, may help us. Let us write C(N+2,R-1) in a triangular mode:

```
C(N-1,R-1) + C(N-1,R-2) + C(N-1,R-3) +... + C(N-1,0) = C(N,R-1)
           + C(N-1,R-2) + C(N-1,R-3) +... + C(N-1,0) = C(N,R-2)
                        + C(N-1,R-3) +... + C(N-1,0) = C(N,R-3)
                                      ... + C(N-1,0) = C(N,R-R+1.)
                                      ... + C(N-1,0) = C(N,0)
                                                     = C(N+1,R-1)
           + C(N-1,R-2) + C(N-1,R-3) +... + C(N-1,0) = C(N,R-2)
                        + C(N-1,R-3) +... + C(N-1,0) = C(N,R-3)
                                      ... + C(N-1,0) = C(N,R-R+1)
                                      ... + C(N-1,0) = C(N,0)
                                                     = C(N+1,R-2)
                        + C(N-1,R-3) +... + C(N-1,0) = C(N,R-3)
                                      ... + C(N-1,0) = C(N,R-R+1)
                                      ... + C(N-1,0) = C(N,0)
                                                     = C(N+1,R-3)
                                      ... + C(N-1,0) = C(N,R-R+1)
                                                     = C(N+1,R-R+1)
                                          + C(N-1,0) = C(N,0)
                                                     = C(N+1,0)
                                                     = C(N+2,R-1)
```

In the first triangle whose sum is C(N+1,R-1), C(N-1,R-1) is repeated once, C(N-1,R-2) twice, and any combinatorial element is repeated J times. Since J is equal to x, C(N+1,R-1) is exactly the sum of x values of C(N,R), and the sum of these elements plus that one for x=0 is equal to C(N,R). Thus, the mean or E(x) is directly C(N+1,R-1)/C(N,R)=(N+R-1)! R!(N-1)!/[(N+R-1)!(R-1)!N!] = R/N as showed in the example (Nº balls/Nº boxes). The $E(x^2)$ is also obtained from the triangles. We remark that C(N-1, R-1) is repeated once in the first triangle. C(N-1,R-2) appears twice in the first triangle and once in the second one. C(N-1,R-3) is repeated three times, twice and once in the first, second and third triangle, respectively. Thus, any combinatorial element corresponding to x (J) is repeated a number of times equal to the sum of natural numbers from 1 to x. This sum is $x(x+1)/2=(x^2 +x)/2$. Thus C(N+2,R-1) is equal to the sum of $(x^2+x)/2$ values of non 0 values of C(N,R). As C(N+1,R-1) is the sum of x values of C(N,R), the expected sum of x^2 $[E(x^2)]$ is [2C(N+2,R-1)-C(N+1,R-1)]/C(N,R). The numerator is {2(N+R)!/[(R-1)!(N+1)!]-(N+R-1)!/[(R-1)!N!]}=(N+R-1)!(N+2R-1)/[(R-1)!(N+1)!], which divided by C(N,R)=(N+R-1)!/[R!(N-1)] yields $E(x^2)=[2R^2+R(N-1)]/[N(N+1)]$. The variance is $E(x^2)-[E(x)]^2=[2R^2+R(N-1)]/[N(N+1)]-(R/N)^2 = V(x)$ = [R(N+R)(N-1)]/[N(N+1)]. We can test the observed distribution of any base in relation to its no-base by these expected parameters we have just calculated. This B-E statistics was also obtained by Gouet and López-Fenner (1986) for the nucleotide distribution in chromosomes, when they evolve by random rearrangements (Valenzuela, 1985). Since the expected mean R/N

is equal to the observed mean, we can test only the variance. This is not a Gaussian distribution that allows an F or χ^2 variance ratio test; thus, variances were tested by a z test with the expected standard error of the variance [SE_V =square root of the variance of the variance (V_V) divided by the number of boxes, N].

$$z = \frac{\text{(Observed variance - Expected variance)}}{\text{square root [(variance of variance)/N]}} = \frac{V_O - V_E}{SE_V}$$

$V_V=E\{[x-E(x)]^2-V(x)\}^2=E(x^4)-4E(x^3)E(x)+8[E(x)]^2E(x^2)-4[E(x)]^4-[E(x^2)]^2$

(see also Wright, 1968; Spiegel et al., 2001). $E(x^3)$ and $E(x^4)$ were obtained by trial and error with B-E matrix elements as it was for E(x) and $E(x^2)$. They resulted to be:

$E(x^3)=[2C(N+4,R-1)-C(N+1,R-1)-2C(N+4,R-4)]/C(N, R)=R[2(N+R)(N+R+1)(N+R+ 2)-(N+1)(N+2)(N+3)-2(R-3)(R-2)(R-1)]/[N(N+1)(N+2)(N+3)] =$

$E(x^3) = R[6R(N+R-1)+(N-1)(N-2)]/[N(N+1)(N+2)]$

$E(x^4)=[4C(N+6,R-1)-3C(N+3,R-1)-4C(N+7,R-3)+3C(N+3,R-3)+4C(N+8,R-4)-4C(N+ 7,R-4)-4C(N+8,R-6)+4C(N+6,R-6)]/C(N,R) =4R[(N+R)(N+R+1)(N+R+2)(N+R+3)(N+R+4)+(R-1)(R-2)(R-3)(R-4)(R-5)-(R-1)(R-2)(N+R)(N+R+1)(N+2R-1)]/[N(N+1)(N+2)(N+3)(N+4)(N+5)]-3R[(N+R)(N+R+1)-(R-1)(R-2)]/[N(N+1)(N+2)]=$

$E(x^4)=R\{(N-1)[(N-1)(N-6)+2R(7N+18R-12)]+24R^3\}/[N(N+1)(N+2)(N+3)].$

The analytical demonstration was not found. Numerical calculi were performed in a 25x25 B-E matrix and agreed with those given by formulae. This agreement was also found for the 8954 bases of HIV-1 and the 2627 bases of S-env DNA segment. The correction by the B-E covariance $[-R(R+N)]/[N^2(N+1)] = -(R/N)[(R/N)+1][1/(N+1)]$ was neglected, because of its small value [near $1/(N+1)$].
