# Supplementary material for: Non-random pre-transcriptional evolution in HIV-1. A refutation of the foundational conditions for neutral evolution
Source: Genet Mol Biol. 2009 Jan 30;32(1):159–69. doi: 10.1590/S1415-47572009005000025 (PMC3032973; doi:10.1590/S1415-47572009005000025)
Supplement: Appendix S2 — Testing Runs. [file gmb-32-1-159-suppl2.pdf]

S2.Testing Runs

Let AAZZAZZZAAAAZZAZ be a sequence of 8 A (N_A=8) and 8 No-A (Z, N_Z=8) with 4 "runs" of A and 4 runs of Z, $N=N_A+N_Z$ =16. The variable "number of runs" (u, this case 8) distributes with mean $m_u=[(2N_AN_Z/N)+1]$, and variance $s_u^2=[2N_AN_Z(2N_AN_Z-N)]/[N^2(N-1)]$. If N_A and N_Z are larger than 9, the sample distribution of u is near Gaussian. Thus, run sequences can be tested by a z test for normal distribution [$z = (u-m_u)/s_u$] (Freund et al., 2000; Spiegel et al., 2001).
